# Noncoding RNAs Associated with PPARs in Etiology of MAFLD as a Novel Approach for Therapeutics Targets

**DOI:** 10.1155/2022/6161694

**Published:** 2022-09-17

**Authors:** Fatemeh Kazeminasab, Maryam Baharlooie, Kamran Ghaedi

**Affiliations:** ^1^Department of Physical Education and Sport Sciences, Faculty of Humanities, University of Kashan, Ravand Street, Kashan 87317-35153, Iran; ^2^Department of Cell and Molecular Biology and Microbiology, Faculty of Biological Science and Technology, University of Isfahan, Hezar Jerib Avenue, Azadi Sq., Isfahan 81746-73441, Iran

## Abstract

**Background:**

Metabolic associated fatty liver disease (MAFLD) is a complex disease that results from the accumulation of fat in the liver. MAFLD is directly associated with obesity, insulin resistance, diabetes, and metabolic syndrome. PPAR*γ* ligands, including pioglitazone, are also used in the management of this disease. Noncoding RNAs play a critical role in various diseases such as diabetes, obesity, and liver diseases including MAFLD. However, there is no adequate knowledge about the translation of using these ncRNAs to the clinics, particularly in MAFLD conditions. The aim of this study was to identify ncRNAs in the etiology of MAFLD as a novel approach to the therapeutic targets.

**Methods:**

We collected human and mouse MAFLD gene expression datasets available in GEO. We performed pathway enrichment analysis of total mRNAs based on KEGG repository data to screen the most potential pathways in the liver of MAFLD human subjects and mice model, and analyzed pathway interconnections via ClueGO. Finally, we screened disease causality of the MAFLD ncRNAs, which were associated with PPARs, and then discussed the role of revealed ncRNAs in PPAR signaling and MAFLD.

**Results:**

We found 127 ncRNAs in MAFLD which 25 out of them were strongly validated before for regulation of PPARs. With a polypharmacology approach, we screened 51 ncRNAs which were causal to a subset of diseases related to MAFLD.

**Conclusion:**

This study revealed a subset of ncRNAs that could help in more clear and guided designation of preclinical and clinical studies to verify the therapeutic application of the revealed ncRNAs by manipulating the PPARs molecular mechanism in MAFLD.

## 1. Introduction

Nonalcoholic fatty liver disease (NAFLD) is a complex disease that results from the accumulation of fat in the liver. In hepatic steatosis, fat in the form of triglycerides and cholesterol esters accumulate in hepatocytes [1]. NAFLD is also considered a “metabolic disease” since it is closely linked with metabolic disorders, including dyslipidemia, obesity, and type 2 diabetes [2]. The new terminology of NAFLD has been updated as metabolic associated fat liver disease (MAFLD), which is used in this review article [3]. MAFLD is one of the most common causes of chronic liver disease, histologically classified as simple steatosis, nonalcoholic steatohepatitis (NASH), fibrosis, cirrhosis, and liver cancer [4]. Prevalence of MAFLD is increasing every year, with 25% of adults worldwide being infected with this disease [5]. MAFLD is directly associated with obesity, insulin resistance (IR), diabetes, and metabolic syndrome [6]. The most important primary risk factors for MAFLD are high cholesterol, obesity, hyperlipidemia, and type 2 diabetes mellitus (T2DM), and other risk factors include hepatitis C and glucocorticoids [7]. Common management of MAFLD is based on the treatment of hepatic metabolic disorders, IR, and lifestyle improvements such as diet and physical activity for weight loss. PPAR*γ* ligands, including thiazolidinediones (TZD), such as pioglitazone and rosiglitazone are also used to treat this disease [8–10].

The cellular and molecular mechanisms involved in steatosis and MAFLD are not yet fully understood. However, evidence suggests that various factors, including inflammation of adipose tissue, hepatic lipogenesis, IR, mitochondrial abnormalities in the liver cells, and oxidative stress are involved in the progression of steatosis to fibrosis and cirrhosis [11–13].

The liver controls fat homeostasis through complex interactions between hormones, transcription factors, and nuclear receptors. In MAFLD, fat is stored as triglycerides due to molecular pathways. Due to an imbalance between lipid absorption and lipid excretion, fat accumulates in hepatocytes. Pathways such as circulating lipid uptake, fatty acid (FA) oxidation, de novo lipogenesis, and fat export in very-low-density lipoprotein (VLDL) are impaired in MAFLD [14].

In recent studies, a group of noncoding RNAs such as long noncoding RNA (lncRNAs), microRNAs (miRNAs), and circular RNAs (circRNAs) has attracted the attention of many researchers due to the regulation of gene transcription. These noncoding RNAs play a critical role in various diseases such as diabetes [15], obesity [16] as well as liver diseases, including MAFLD [17–19].

lncRNAs as a subgroup of ncRNAs with more than 200 nt lengths that modulate the post-transcriptional stages of genes of degradation, splicing, and translation of target genes [20]. LncRNAs also play a key role in regulating epigenetics and gene transcription [21]. The other subgroup of ncRNAs stand small noncoding RNAs consisting of miRNAs and circular RNAs, which also take part in the regulation of target gene expression. miRNAs, mainly suppress gene expression by binding to the 3′-UTR of target mRNAs. On the other hand, circRNAs are known particularly to sponge miRNAs and protect target mRNAs, as lncRNAs also do. Nonetheless, inspecting these ncRNAs function as a network would help us better understand their roles and impact on a particular gene expression phenomenon. The aim of this study was to identify noncoding RNAs, including miRNAs, lncRNAs, and circRNAs in the etiology of MAFLD as novel approaches for therapeutic targets.

## 2. Methods

### 2.1. Bioinformatics Screening and Analysis

Articles were searched on PubMed and Google Scholar from 2010 to 2022. The search was performed with the keyword fatty liver disease. We collected human and mouse fatty liver disease gene expression datasets available in Gene Expression Omnibus (GEO) (Table 1), and reanalyzed them to examine and compare them with the pathways. Gene expression data were revealed from literature to explore novel potential interconnections for further studies. We selected datasets regarding human fatty liver disease subjects and mice treated with a high-fat diet developing fatty liver disease. Limma package was used in this tool and the significance threshold of expression changes were indicated with *p*-value<0.05 in the Benjamini & Hochberg test (false discovery rate).

### 2.2. Pathway Enrichment and Network ClueGO Analysis

**Figure 1:**
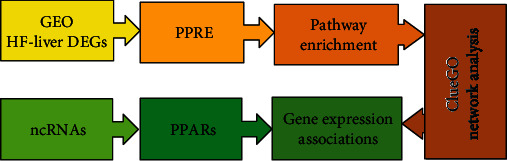
Flowchart of the study.

We performed pathway enrichment analysis of total mRNAs using Database for Annotation, Visualization, and Integrated Discovery (DAVID) *v*6.8 (https://david.ncifcrf.gov/) based on Kyoto Encyclopedia of Genes and Genomes (KEGG) repository data to screen the most potential pathways in NAFLD mice liver. Then, we constructed and visualized the network via the ClueGO app, along with analyzing for protein-protein interactions by Cytoscape software, using interaction scores from STRING DB *v*11.0 set the threshold to 0.4 interaction score. The flowchart of the study is shown in Figure 1.

### 2.3. ncRNA-mRNA Collection and Direct Interactions Prediction

In this study, data mining and bioinformatics analyses were used to evaluate if noncoding RNAs associated to MAFLD also target PPARs. We collected and integrated a list of strongly validated associations of ncRNAs and MAFLD. Then, LncRRIsearch database was used to analyze data of lncRNA-mRNA interaction prediction [22]. With a binding energy threshold of -12 kcal/mol, we queried lncRNAs list to collect direct mRNA targets. We chose this threshold since we preferred to have a wide range of mRNAs for pathway enrichment analysis. Then in order to get an insight to the functions of these mRNA sets, we enriched each total target list in Enrich R tool.

### 2.4. Disease-Causality Screening

Finally, we screened disease associations of the common ncRNAs between MAFLD and PPARs using HMDD v3.2 (https://www.cuilab.cn/hmdd) and LncRNADisease *v*2.0 (http://www.rnanut.net/lncrnadisease). We set screening criteria including experimental detection method, strong validation assays (such as ChIP, qPCR, Western blot, Luciferase reporter gene assay, Northern blot, and RNAi,), a score of 0.5 to 1.0, and excluded noncausal associations. These results could help in more clear and guided designation of preclinical and clinical studies to verify the therapeutic application of the revealed ncRNAs.

### 2.5. Interaction Network Construction and Analysis

Finally, the resulted causal ncRNAs were used to construct a network for representation of their interactions with the analyzed genes of fatty liver disease. We collected all mRNAs interacting with the causal ncRNAs by the seed region complementarity algorithm of miRNAs-mRNAs interactions in TargetScan Human 7.0 database, CircRNAs-mRNAs interactions in Circ-Interactome (https://circinteractome.nia.nih.gov/), and lncRNAs-mRNAs interactions in LncRRIsearch databases. We selected a subset of top 100 interactions among those that showed above 75% of total score. Then, we applied CytoScape 3.6.1 software to construct and analyze the interactions network based on Degree Centrality parameter.

## 3. Results

### 3.1. Genes Screening in GEO

We screened mRNAs from datasets GSE63067 and GSE85439, with differential expression in the liver using Limma package in GEO2R tool, based on criteria of log FC > 0.5, and adj *p*-value≤0.05 for human patients vs. controls, and log FC > 2, and adj *p*-value≤0.05 for mouse high-fat diet (HF) compared to low-fat (LF) diet groups. Moreover, GEO data analysis revealed 444 differentially expressed genes (DEGs) in human MAFLD, as well as 350 DEGs in mice MAFLD.

### 3.2. Pathway Enrichment and ClueGO Analysis

**Figure 2:**
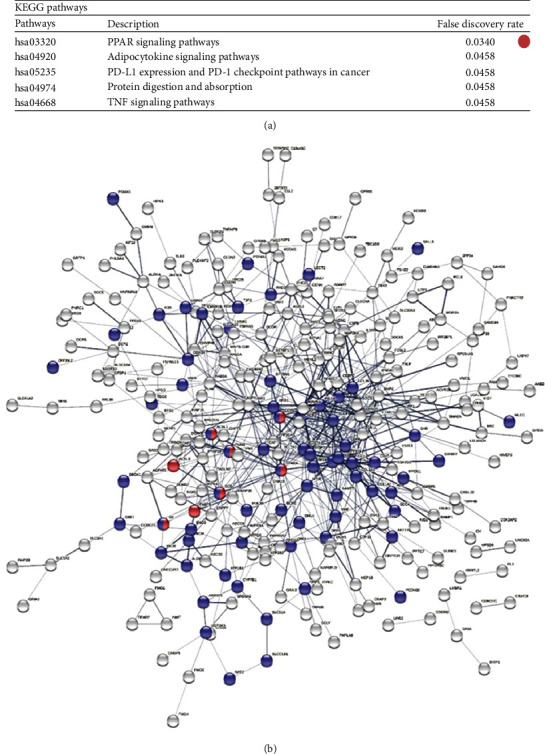
KEGG pathway enrichment and protein-protein network of human MAFLD DEGs. (a) Top 5 enriched pathways with significant FDR score and (b) protein-protein network illustrating PPARs signaling genes as red and liver-expressed genes as blue nodes.

The most powerfully enriched KEGG pathway in the human MAFLD data was PPAR signaling pathway. Also, this pathway was significantly enriched in mouse MAFLD data, demonstrating its significance in MAFLD molecular pathobiology. The top three KEGG pathways enriched in human MAFLD data included hsa03320 PPAR signaling pathway, FDR = 0.034; hsa04668 TNF signaling pathway, FDR = 0.0458; and followed by hsa04920 the adipocytokine signaling pathway, FDR = 0.0458 which are all associated with the pathobiology of MAFLD.  Figure 2 shows KEGG pathway enrichment and protein-protein network of human MAFLD DEGs. Also, Supplementary Figure 1 shows KEGG pathway enrichment and protein-protein network of mouse MAFLD DEGs.

**Figure 3:**
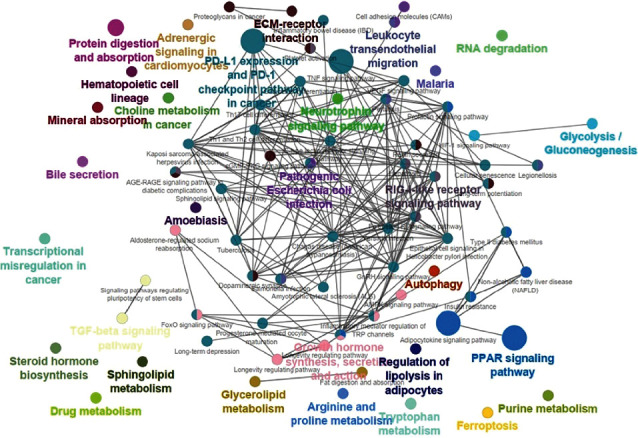
ClueGO results of interconnection between pathways in the network of human MAFLD DEGs. Hub pathways are shown as bigger nodes.

**Figure 4:**
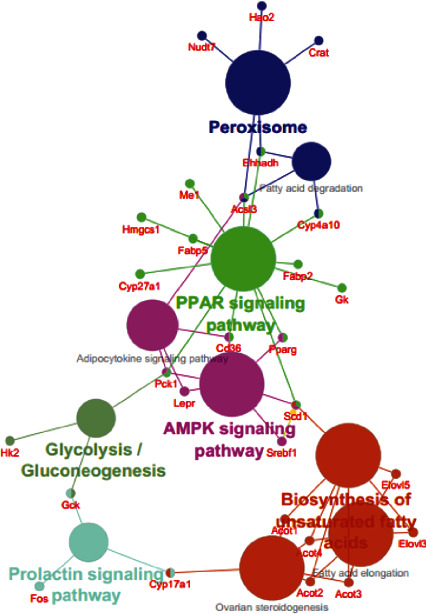
ClueGO results of interconnection between pathways in the network of mouse MAFLD DEGs. Hub pathways are shown as bigger nodes.

Nevertheless, ClueGO analysis results of human data revealed that pathways corresponding to adipocytokine signaling pathway, insulin resistance, MAFLD, and T2DM have significant association via an Ontology gene connection with the PPAR signaling pathway as a hub node in the ClueGO pathway. Figures 3 and 4 are shown as ClueGO results of interconnection between pathways in the network of NAFLD DEGs in humans and mice, respectively.

Moreover, ClueGO analysis results of mouse data revealed that pathways corresponding to peroxisome, AMPK signaling, Glycolysis/Gluconeogenesis, Prolactin signaling, and Biosynthesis of unsaturated FAs have major association via an Ontology gene connection with the central axis of the PPAR signaling pathway.

### 3.3. Noncoding RNA Associated with MAFLD

A list of noncoding RNA associated with MAFLD were identified as 79 miRNAs (Supplementary Table 1), 32 lncRNAs (Supplementary Table 2), and 16 circRNAs (Supplementary Table 3) associated with fatty liver were listed. The lncRNAs-related pathways and genes are shown in Table 2.

**Figure 5:**
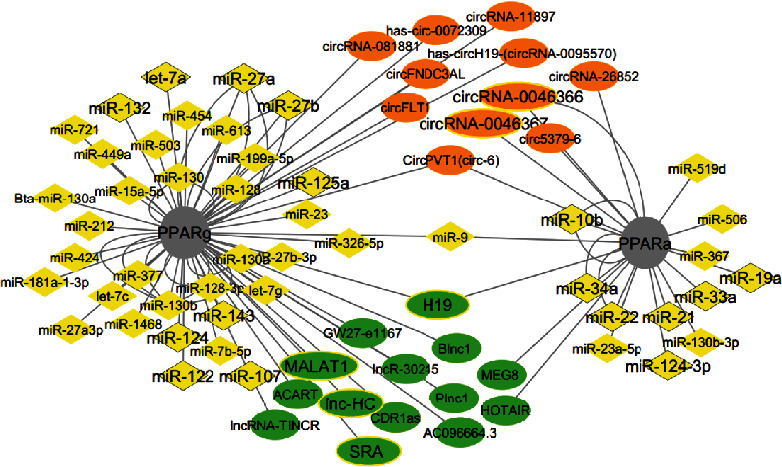
Strongly validated association of ncRNAs and PPARs. ncRNAs that are involved in MAFLD pathophysiology are depicted as bold titles with bordered nodes.

Moreover, common noncoding RNAs of MAFLD and PPARs were identified in this study.  Figure 5 shows strongly validated associations between ncRNAs and PPARs. Subsequently, they were subjected to assess disease causality features by bioinformatics tools.

### 3.4. Causal ncRNAs Associated with MAFLD and PPARs

Table 3 shows causal lncRNAs and circRNAs associated with fatty liver disease and PPARs, and Table 4 shows causal miRNAs related to fatty liver disease and PPARs. These miRNAs are validated by strong methods.

### 3.5. Causal ncRNAs-Fatty Liver mRNAs Interaction Network

**Figure 6:**
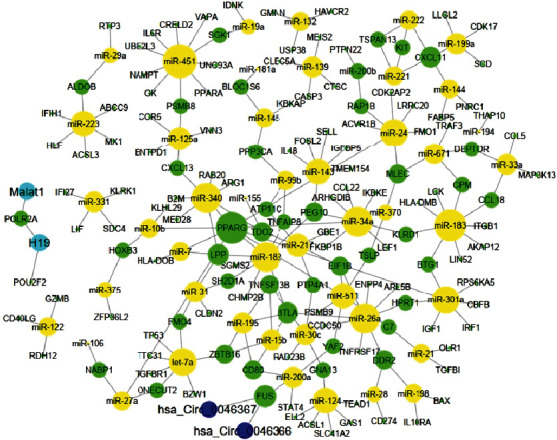
Interaction network construction and analysis of causal ncRNAs-fatty liver mRNAs. Hub genes and hub ncRNAs based on degree centrality parameter are showed as bigger nodes. MiRNAs, mRNAs, circular RNAs, and lncRNAs are depicted as yellow, green, blue, and turquoise nodes, respectively.

A network consisted of 191 Nodes and 203 Edges was constructed. Hub genes and hub ncRNAs based on degree centrality parameter were shown as bigger nodes. MiRNAs, mRNAs, circular RNAs, and lncRNAs are depicted as yellow, green, blue, and turquoise nodes, respectively (Figure 6).

## 4. Discussion

Retrospective literature mining provided evidence for association of PPARs with pathways of MAFLD. Recently, many efforts have been made to identify MAFLD genes and ncRNAs. Correspondingly, the importance of the PPAR signaling pathway has been recognized and discussed widely. In this pathway, PPAR*γ* and PPAR*α* play significant roles in MAFLD development and alleviation. In agreement with previous studies, we identified the PPAR signaling pathways as a hub pathway in MAFLD gene expression data.

Adipocytokines, including tumor necrosis factor-*α* (TNF-*α*), resistin, leptin, and adiponectin are secreted from adipose tissue. These molecules act as chemokines for macrophages to accumulate in the adipose tissue. Accordingly, obesity makes a chronic low-grade inflammatory condition that is considered a risk factor for liver diseases [23]. Adipocytokine dysregulation also leads to a decrease in insulin resistance and an increase in proinflammatory cytokines.

Recent studies revealed that there is a crosstalk between adipocytokine and PPAR signaling, in such a way that hepatic lipo-inflammation is modulated by adiponectin-activated PPARs and PPAR-induced adiponectin. The PPAR*α* and PPAR*γ* vastly function to reduce inflammation, while PPAR-beta(*β*) is a potential target for the treatment of insulin resistance [24].

Adiponectin activates hepatic adiponectin Receptor 2, then downstream AMP-activated protein kinase (AMPK) pathway is initiated, and triggers PPAR transcription factors. This results in the upregulation of genes responsible for ameliorating oxidative status, inflammation, and high levels of triglycerides [25]. Furthermore, adiponectin is one of the upregulated genes by PPAR*γ* agonists, rosiglitazone, and pioglitazone, which are reported to improve IR in diabetic patients [26]. PPAR*γ* in adipocytes modifies target genes involved in fat uptake, lipid storage, the release of insulin-sensitive adipokines, and the production of inflammatory cytokines. PPAR*γ* activation increases fat storage in adipose tissue and enhances insulin sensitivity [27]. The researchers showed that knockdown PPAR*γ* in obese *ob/ob* mice reduced hepatic triglyceride contents compared to control mice. In this condition, excess fat delivery to other tissues, including the striated muscles, leads to increased insulin resistance and the development of T2DM [28]. On the other hand, studies have reported that PPAR*γ* expression in the liver of patients with MAFLD increases and activates the expression of adipogenic genes and exacerbates hepatic steatosis. It is noteworthy that clinical studies have shown that treatment of fatty liver with TZD reduces hepatic steatosis. The reduction in steatosis is due to the effects of TZD on adipose tissue, which prevents excess body fat from entering the liver and prevents the formation of dysfunctional fat cells. Increased adipose tissue formation following TZD is seen with weight gain in patients with TZ treated with fatty liver [27].

PPAR*α* is expressed in tissues such as the liver and controls FA transport, hepatic glucose production, and FA metabolism. PPAR*α* is a nutritional sensor that controls lipid metabolism in response to feeding and fasting [29]. PPAR*α* regulates the transcription of genes involved in beta-oxidation, FA transport, and gluconeogenesis [30]. It also negatively regulates proinflammatory signaling pathways and plays a vital role in fatty liver disease. It is noteworthy that PPAR*α* activation improves inflammation, fibrosis, and hepatic steatosis in MAFLD model mice. Therefore, PPAR*α* agonists and their modulators are used as a strategy for the management and treatment of fatty liver [31].

In this study, we also focused on the ncRNA network regulating the PPAR pathway. Interestingly, several ncRNAs associated with MAFLD have been validated by qRT-PCR for regulation of PPAR*γ* and PPAR*α* before. Nevertheless, we constructed and analyzed a network consisting of liver disease causal ncRNAs interacting with MAFLD mRNAs.

## 5. Conclusion

MAFLD is a prevalent disorder and refers to a group of conditions where there is an accumulation of excess fat in the liver. MAFLD is directly associated with obesity, metabolic syndrome, and diabetes mellitus. Noncoding RNAs play a critical role in several diseases such as diabetes, obesity, and liver diseases, including MAFLD. The results of this study show that a subset of causal noncoding RNAs associated with PPARs can be used to treat MAFLD. This study could help in more clear and guided designation of preclinical and clinical studies to verify the therapeutic application of the revealed ncRNAs.

## Figures and Tables

**Table 1:** The differentially expressed gene (DEG) for all employed Datasets in the present study.

GEO accession	GEO platform	Tissue	Organism	Characteristics of subject	Case samples	Control samples	Up Reg. Genes	Down Reg. Genes
GSE63067 [32]	GPL570	Liver samples	Homo sapiens	Steatosis and NASH	11	7	552	481
GSE85439 [33]	GPL15691	Liver samples	Mus musculus (C57BL/6 ob/ob mice)	Mice fed HFD for 12 weeks.	10	9	880	565

HFD (High-fat diet): 60% kcal from fat, 20% kcal from protein, and 20% kcal from carbohydrate.

**Table 2:** Pathways and genes associated with lncRNAs.

LncRNAs	Pathways (genes involved with pathways)
H19	Lysine degradation (KMT2D; SETD1B; KMT2B; SETD1A) Ras signaling pathway (SYNGAP1; IGF2; KSR2; GNB5; GRIN2B) IL-17 signaling pathway (MUC5B; MUC5AC) Circadian entrainment (GNB5; GRIN2B)
NEAT1	Lysine degradation (KMT2D; SETD1B; SETD1A) Circadian entrainment (KCNJ6; GNB5; GRIN2B) Ras signaling pathway (SYNGAP1; IGF2; KSR2; GNB5; GRIN2B) Dopaminergic synapse (KCNJ6; GNB5; GRIN2B) Maturity onset diabetes of the young (PDX1) Peroxisome (MLYCD; PEX26)
MEG3	GABAergic synapse (GABBR1; GAD2; CACNA1F) Beta-alanine metabolism (GAD2; MLYCD) GnRH secretion (GABBR1; CACNA1F) Arrhythmogenic right ventricular cardiomyopathy (CACNG8; CACNA1F) Taurine and hypotaurine metabolism (GAD2)
HULC	Ras signaling pathway (FGF17; SYNGAP1; GNB5; GNG13) Retrograde endocannabinoid signaling (NDUFA10; GNB5; GNG13) Gastric cancer (FGF17; MUC2; WNT7B)
FTX	Herpes simplex virus 1 infection (ZNF891; ZNF383; ZNF382; ZNF8; ZNF587; ZNF26; ZNF850) Chagas disease (SMAD2; GNAI3; CFLAR) Bladder cancer (RPS6KA5; MDM2) Glycosaminoglycan biosynthesis (CHST12; ST3GAL2) Signaling pathways regulating pluripotency of stem cells (SMAD2; NANOG; BMPR1A) Hippo signaling pathway (SMAD2; WTIP; BMPR1A) p53 signaling pathway (MDM2; MDM4)
MALAT1	IL-17 signaling pathway (MUC5B; MUC5AC) Ras signaling pathway (SYNGAP1; KSR2; GNB5)
MAYA	Beta-alanine metabolism (GAD2; MLYCD) Focal adhesion (MYL5; RAP1A; PARVG; ITGA2) Leukocyte transendothelial migration (MYL5; RAP1A; MAPK13) Regulation of actin cytoskeleton (NCKAP1; MYL5; ABI2; ITGA2) Platelet activation (RAP1A; ITGA2; MAPK13) Ras signaling pathway (SYNGAP1; RAP1A; IGF2; REL) Cellular senescence (CDKN2A; IPK2; MAPK13) Taurine and hypotaurine metabolism (GAD2)
AC012668	Spinocerebellar ataxia (ATXN3; PSMD11; GRIN2B) Huntington disease (PSMD11; POLR2A; SDHC; GRIN2B)
CCAT1	Bladder cancer (CDKN2A; MDM2) Cell cycle (SMAD2; CDKN2A; MDM2) Cellular senescence (SMAD2; CDKN2A; MDM2) Hepatocellular carcinoma (SMAD2; CDKN2A; IGF2) Melanoma (CDKN2A; MDM2) p53 signaling pathway (CDKN2A; DM2) Glioma (CDKN2A; MDM2) Chronic myeloid leukemia (CDKN2A; MDM2) Pancreatic cancer (SMAD2; CDKN2A)
SNHG20	Maturity onset diabetes of the young (HNF1A)
GM10804	Phototransduction (SLC24A1; CNGB1) Circadian entrainment (RYR1; GRIN2A; GRIN2B) Nicotine addiction (GRIN2A; GRIN2B) Glutamatergic synapse (GRIN2A; GRIN2B; SHANK1) cAMP signaling pathway (GRIN2A; ATP2B4; GRIN2B; CNGB1) Cocaine addiction (GRIN2A; GRIN2B) Systemic lupus erythematosus (GRIN2A; CD80; GRIN2B) Spinocerebellar ataxia (RYR1; GRIN2A; GRIN2B) Long-term potentiation (GRIN2A; GRIN2B) Amphetamine addiction (GRIN2A; GRIN2B)
GAS5	Ras signaling pathway (SYNGAP1; IGF2; KSR2; GNB5) Glycerolipid metabolism (GLYCTK; LIPG)
CTCFLOS	Cocaine addiction (GRIN2A; DLG4; GRIN2B) Glutamatergic synapse (GRIN2A; DLG4; GRIN2B; SHANK1) Nicotine addiction (GRIN2A; GRIN2B) Systemic lupus erythematosus (GRIN2A; CD80; GRIN2B) Long-term potentiation (GRIN2A; GRIN2B) Amphetamine addiction (GRIN2A; GRIN2B)
RP11-484 N16.1	Spliceosome (HNRNPA3; FUS; PRPF40B; HNRNPA1) Amyotrophic lateral sclerosis (HNRNPA3; FUS; NEFM; HNRNPA1; GRIN2B) Arrhythmogenic right ventricular cardiomyopathy (CACNG7; CACNG8)
PLATR4	Gastric acid secretion (KCNJ15; MYLK4)

**Table 3:** Causal lncRNAs and circRNAs associated with fatty liver disease and PPARs.

Causal ncRNAs	Interconnected disease with MAFLD	Strong evidence
H19	Hepatocellular carcinoma	Northern blot/qPCR/RIP/western blot
MALAT1	Hepatocellular carcinoma	Apoptosis assay/Luciferase reporter gene assay/metastasis assay/qPCR/qRT-PCR/transwell assay/western blot/wound healing assay
	Fatty liver disease	qPCR/RIP/western blot
CircRNA_0046366	Fatty liver disease	Luciferase reporter gene assay/qPCR/western blot
CircRNA_0046367	Fatty liver disease	Luciferase reporter gene assay/qPCR/western blot

**Table 4:** Causal miRNAs associated with fatty liver disease and PPARs.

Causal miRNAs	Interconnected disease with MAFLD
miR-29a	Hepatocellular carcinoma
miR-122	Hepatocellular carcinoma
miR-21	MAFLD, hepatocellular carcinoma
miR-34a	Liver diseases, MAFLD
miR-451	MAFLD, hepatocellular carcinoma
miR-33a	Fatty liver disease
miR-132	Hepatocellular carcinoma
miR-181a	Hepatocellular carcinoma
miR-221	Fatty liver disease, hepatocellular carcinoma
miR-222	Fatty liver disease
miR-375	Fatty liver disease, hepatocellular carcinoma
miR-26a	Fatty liver disease, hepatocellular carcinoma
miR-139	Hepatocellular carcinoma, MAFLD
miR-340	Hepatocellular carcinoma
miR-125a	Hepatocellular carcinoma
miR-182	Hepatocellular carcinoma, liver injury
miR-155	Hepatocellular carcinoma
miR-143	Hepatocellular carcinoma
miR-370	Hepatocellular carcinoma, liver cirrhosis
miR-200a	Liver cirrhosis
miR-200b	Hepatocellular carcinoma
miR-99b	Hepatocellular carcinoma
miR-27a	MAFLD, hepatocellular carcinoma
miR-30C	MAFLD
miR-15b	Liver failure
miR-19a	Hepatocellular carcinoma
miR-331	Hepatocellular carcinoma
miR-24	Hepatocellular carcinoma
miR-199a	Hepatocellular carcinoma
miR-145	Hepatocellular carcinoma
miR-223	Hepatocellular carcinoma
miR-144	MAFLD, hepatocellular carcinoma
miR-7	Hepatocellular carcinoma
miR-28	Hepatocellular carcinoma
miR-511	Hepatocellular carcinoma
miR-671	Hepatocellular carcinoma
miR-195	Hepatocellular carcinoma
miR-31	Liver cancer, hepatocellular carcinoma
miR-194	Hepatocellular carcinoma
miR-183	Hepatocellular carcinoma
miR-10b	Hepatocellular carcinoma
let-7a	Hepatocellular carcinoma
miR-217	Hepatocellular carcinoma
miR-106	Hepatocellular carcinoma
miR-124	Hepatocellular carcinoma
miR-301a	Hepatocellular carcinoma
miR-198	Hepatocellular carcinoma

## Data Availability

All data generated or analyzed during this study are available upon request.

## Abbreviations

MAFLD: Metabolic associated fatty liver disease
NAFLD: Nonalcoholic fatty liver disease
NASH: Nonalcoholic steatohepatitis
IR: Insulin resistance
TZD: Thiazolidinediones
PPAR*γ*: Peroxisome proliferator-activated receptor gamma
PPAR*α*: Peroxisome proliferator-activated receptor alpha
VLDL: Very-low-density lipoprotein
lncRNAs: Long noncoding RNA
miRNAs: microRNAs
circRNAs: Circular RNAs
FA: Fatty acid
GEO: Gene Expression Omnibus
HF: High-fat diet
LF: Low-fat diet
DAVID: Database for Annotation, Visualization, and Integrated Discovery
KEGG: Kyoto Encyclopedia of Genes and Genomes
T2DM: Type 2 diabetes mellitus
TNF-*α*: Tumor necrosis factor-*α*
AMPK: AMP-activated protein kinase
HCC: Hepatocellular carcinoma.

## Supplementary Materials

Supplementary Materials Supplementary Figure 1: KEGG pathway enrichment and protein-protein network of mouse NAFLD DEGs. A. Top 4 enriched pathways with significant FDR score. B. protein-protein network illustrating PPARs signaling genes as red, metabolic pathway genes as blue, insulin signaling pathway genes as green, and glycolysis/gluconeogenesis genes as yellow nodes. Supplementary Table 1: the summary of fatty liver disease-related miRNAs [34-58]. Supplementary Table 2: the summary of fatty liver disease-related lncRNAs [15, 58–87]. Supplementary Table 3: the summary of fatty liver disease-related circRNAs [88-99].
