# Supplementary material for: Noncoding RNAs Associated with PPARs in Etiology of MAFLD as a Novel Approach for Therapeutics Targets
Source: PPAR Res. 2022 Sep 17;2022:6161694. doi: 10.1155/2022/6161694 (PMC9509273; doi:10.1155/2022/6161694)
Supplement: Supplementary Materials — Supplementary Figure 1: KEGG pathway enrichment and protein-protein network of mouse NAFLD DEGs. A. Top 4 enriched pathways with significant FDR score. B. protein-protein network illustrating PPARs signaling genes as red, metabolic pathway genes as blue, insulin signaling pathway genes as green, and glycolysis/gluconeogenesis genes as yellow nodes. Supplementary Table 1: the summary of fatty liver disease-related miRNAs [34-58]. Supplementary Table 2: the summary of fatty liver disease-related lncRNAs [15, 58–87]. Supplementary Table 3: the summary of fatty liver disease-related circRNAs [88-99]. [file 6161694.f1.zip › supplemental Fig 1.pptx]

## Slide 1
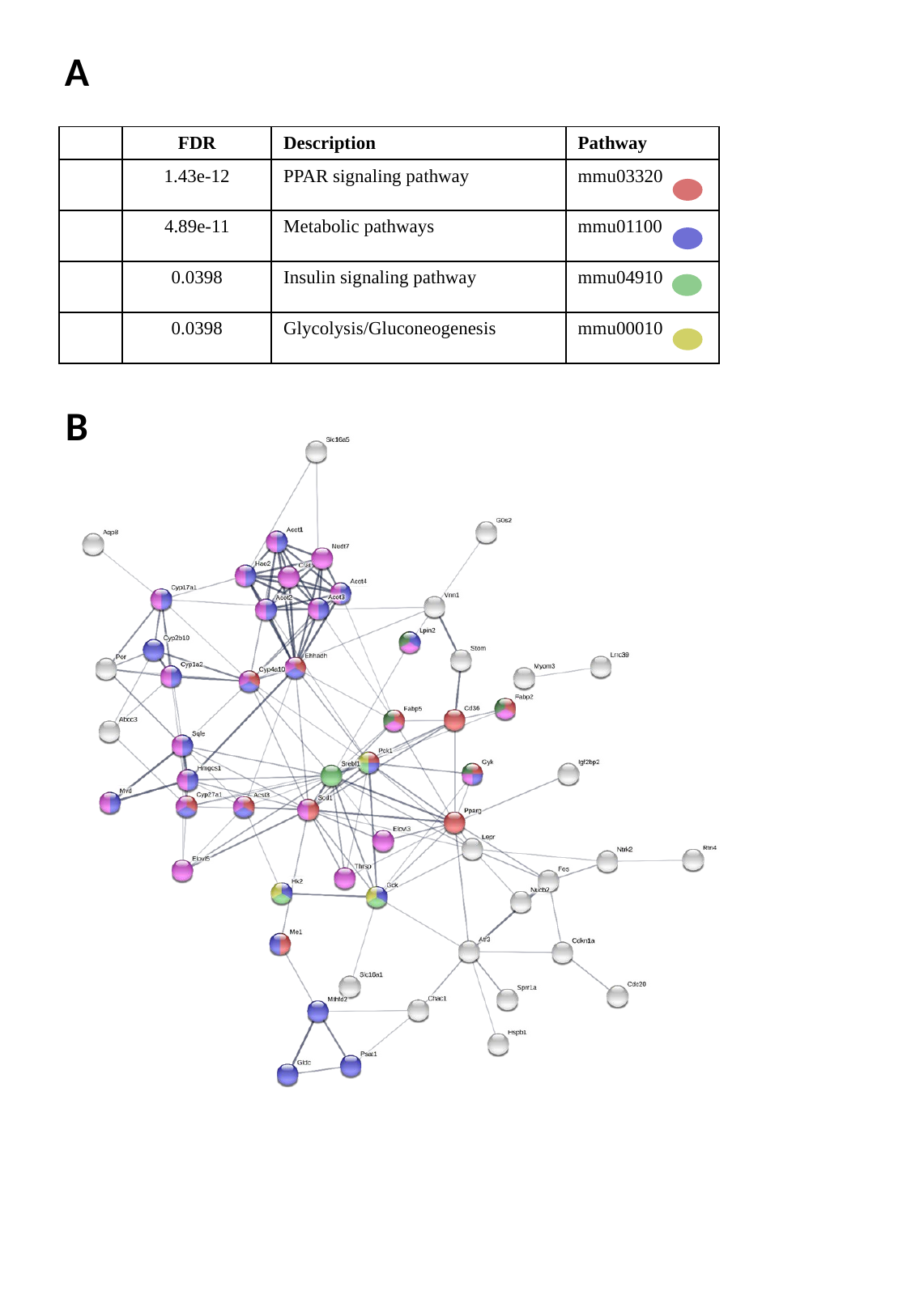

A
| | FDR | Description | Pathway |
| --- | --- | --- | --- |
| | 1.43e-12 | PPAR signaling pathway | mmu03320 |
| | 4.89e-11 | Metabolic pathways | mmu01100 |
| | 0.0398 | Insulin signaling pathway | mmu04910 |
| | 0.0398 | Glycolysis/Gluconeogenesis | mmu00010 |
B
