# Supplementary material for: Noncoding RNAs Associated with PPARs in Etiology of MAFLD as a Novel Approach for Therapeutics Targets
Source: PPAR Res. 2022 Sep 17;2022:6161694. doi: 10.1155/2022/6161694 (PMC9509273; doi:10.1155/2022/6161694)
Supplement: Supplementary Materials — Supplementary Figure 1: KEGG pathway enrichment and protein-protein network of mouse NAFLD DEGs. A. Top 4 enriched pathways with significant FDR score. B. protein-protein network illustrating PPARs signaling genes as red, metabolic pathway genes as blue, insulin signaling pathway genes as green, and glycolysis/gluconeogenesis genes as yellow nodes. Supplementary Table 1: the summary of fatty liver disease-related miRNAs [34-58]. Supplementary Table 2: the summary of fatty liver disease-related lncRNAs [15, 58–87]. Supplementary Table 3: the summary of fatty liver disease-related circRNAs [88-99]. [file 6161694.f1.zip › Supplemental tables.docx]

**Supplementary table 1.** The summary of fatty liver disease-related miRNAs

| **Upregulated miRNAs** | **Downregulated miRNAs** | **References** |
| --- | --- | --- |
| miR-29a, miR-122, miR-21, miR-34a, miR-33a/miR33-b, miR-103/107, miR-181a, miR-192, miR-221/22, miR-375 | miR-132, miR-146b, miR-181d, miR-197 | [34] |
| - | miR-26a | [35] |
| miR-103 | - | [36] |
| miR-33, miR-34a, miR-21, miR-192, miR-146b, miR-221/222, miR-132, miR-181b | miR-122, miR-375, miR-181d, miR-139, miR-197, miR-29a, miR-422 | [37] |
| miR-484, miR-574-3p, miR-125a-5p and miR-182, miR-21, miR-155, miR-23a, miR-143, miR-155, miR-370, miR-34a, miR-99a/b, miR-221/222, miR-16 | miR-340-5p, miR-720, miR-122, miR-200a/b, miR-200c, miR-99b | [38] |
| miR-27a | - | [39] |
| miR-34a, miR-192, miR -27b, miR-22, miR-21, miR-16, miR-451a, miR-27b | miR-197 | [40] |
| miR-34a, miR-192, miR -27b, miR-21, miR-197, miR-30c, miR-16 | - | [41] |
| miR-34a | miR-122, miR-145 | [42] |
| miR-21 | - | [43] |
| miR-122, miR-124, miR-133a, miR-34a, miR-16, miR-451, miR-21 |  | [44] |
| miR-885 | - | [45] |
| miR-19a, miR-19b, miR-125b, miR-375 | - | [46] |
| miR-1290, miR-27b, miR-192, miR-192-5p, miR-27b-3p, | - | [47] |
| miR-223 | - | [48] |
| miR-122, miR-192, miR-19a/b, miR-125b, miR-375, miR-34a, miR-16, miR-451, miR-21, miR-375, miR-125b, miR-33a/b, miR-34a, miR-451, miR-155, miR-221, miR-222, miR-155, miR-16, miR-33a/b, miR-451 | miR-15, miR-16 | [49] |
| miR-497, miR-195, miR-322 | - | [50] |
| miR-31, miR-103, miR-107, miR-194, miR-335-5p, miR-221, miR-200a, miR-182, miR-183, miR-199a-3p, miR-705, miR-1224, miR-16, miR-33, miR-21, miR-221, miR-222, miR-181a/b, let-7a/a, and miR-10b | miR-29c, miR-451, miR-21 | [51] |
| miR-21, miR-216a/217, miR-21, miR-106 | miR-195, miR-122 | [52] |
| miR-122, miR-34a, miR-16, miR-301a-3p | miR-197, miR-146b, miR-181d, miR-99a, miR-375 | [53] |
| miR-33 | - | [54] |
| miR-155, miR-34a, miR-21, miR-221, miR-222, miR-192, miR-19a/b, miR-125b, miR-375, miR-15b, miR-155, miR-223-3p, miR-146b-5p, miR-24, miR-33a/b, miR-335-5p, miR-29, miR-23a, miR-143, miR-1290, miR-10b, miR-370, miR-24, miR-149, miR-27b-3p, miR-148a-3p, miR-99a-5p, miR-192-5p | miR-198, miR-451, miR-122, miR-198, miR-451, miR-144-3p, miR-216, miR-302a, miR-467b, miR-199a-3p | [55] |
| miR-103, miR-107, miR-106b, miRPlus-I137, miR-892a, miR-1282, miR-3663-5p, miR-3924, miR-146b-5p, miR-103, miR-107, miR-155, miR-192 | miR-139-5p, miR-30b-5p, miR-122-5p and miR-422a, miR-29a, miR-29c | [56] |
| - | miR-223 | [57] |
| - | miR-130a | [58] |

**Supplementary table 2.** The summary of fatty liver disease-related lncRNAs

| **LncRNAs** | **Expression levels (up/down)** | **References** |
| --- | --- | --- |
| H19 | up | [58] |
| NEAT1 | up | [59-63] |
| AK012226 | up | [64] |
| MEG3 | down | [65] |
| HULC | up | [66] |
| GM15622 | up | [67] |
| NONMMUT010685 | down | [68] |
| NONMMUT050689 | down | [68] |
| TTC39AOS1 | up | [68] |
| FTX | down | [69] |
| MALAT1 | up | [70] |
| MAYA | up | [71] |
| FLRL2 | down | [72] |
| GM9795 | up | [73] |
| lncARSR | up | [74, 75] |
| Lnc-SPARCL1-1:2 | up | [76] |
| SRA | up | [77] |
| GM12664–001 | down | [78] |
| AC012668 | down | [79] |
| CCAT1 | up | [80] |
| SNHG20 | down | [81] |
| GM10804 | up | [82] |
| GAS5 | up | [83] |
| MIRT2 | down | [84] |
| GM38501 | up | [15] |
| CTCFLOS | up | [15] |
| GM36691 | up | [15] |
| GM44502 | down | [15] |
| lnc-HC | down | [85] |
| RP11-484N16.1 | up | [86] |
| PLATR4 | up | [87] |

**Supplementary table 3.** The summary of fatty liver disease-related circRNAs

| **circRNAs** | **Expression levels** | **References** |
| --- | --- | --- |
| circRNA_021412 | down | [88] |
| circRNA_0046366 | down | [89] |
| circRNA_0046367 | down | [90] |
| mmu-circRNA-0015959  mmu-circRNA-0010514  82671084-82674582 | down  up  down | [91] |
| circScd1 | down | [92] |
| circRNA_002581 | up | [93] |
| circRNA_29981 | up | [94] |
| circRNA_007585  circRNA_002581 | up  up | [95] |
| circRNA_0067835 | up | [96] |
| circRNA_circFBXW4 | down | [97] |
| hsa_circRNA_0048179 | down | [98] |
| circRNA_0057558 | up | [99] |
| circRNA_002581 | up | [93] |
